# Defining Success in Ptosis Surgery: Outcome Heterogeneity and Priorities for Standardization

**DOI:** 10.3390/jcm15197433

**Published:** 2026-09-24

**Authors:** Mattan Arazi, Guy Ben Simon, Ofira Zloto, Ayelet Priel, Mor Schlesinger, Daphna Landau-Prat

**Affiliations:** 1 Department of Ophthalmology, Goldschleger Eye Institute, Sheba Medical Center, Ramat Gan 5265601, Israel; 2 Gray Faculty of Medicine, Tel Aviv University, Tel Aviv 6997801, Israel; 3 Department of Ophthalmology, Kaplan Medical Center, Faculty of Medicine, Hebrew University of Jerusalem, Jerusalem 9190501, Israel

**Keywords:** ptosis surgery, blepharoptosis, surgical outcomes, outcome measures, MRD1, patient-reported outcomes, core outcome set, oculoplastics

## Abstract

Ptosis surgery aims to restore eyelid position, visual function, facial expression, and, in children, visual development. Yet reported success rates reflect how success is defined as much as what surgery achieves. Studies use different eyelid-height thresholds, symmetry criteria, composite definitions, and follow-up intervals, limiting comparison across procedures and patient populations. This narrative review examines outcome assessment after ptosis surgery across six domains: anatomical, functional, aesthetic, patient-reported, safety, and durability outcomes. Margin reflex distance 1 remains the dominant anatomical endpoint, but accepted postoperative values vary and are inconsistently combined with eyelid contour, symmetry, and crease position. Within the same cohort, adding contour and symmetry requirements can reduce the reported success rate. Dynamic function, frontalis compensation, lagophthalmos, and dry eye are also inconsistently defined or omitted, despite their clinical relevance and procedure-specific effects. Anatomical correction does not reliably predict patient-perceived benefit, and no validated ptosis-specific patient-reported outcome measure exists. In children, success depends on visual development, amblyopia prevention, and ocular surface safety rather than eyelid height alone. Most studies assess outcomes within six months, limiting evaluation of recurrence and durability. A core outcome set could improve comparability without imposing a single definition of success across all ptosis subtypes, procedures, and patient populations.

## 1. Introduction

Blepharoptosis is one of the most common eyelid disorders encountered in oculoplastic practice and can affect visual function, appearance, and quality of life [1]. Upper eyelid descent restricts the superior visual field, provokes compensatory brow elevation, and contributes to ocular discomfort and fatigue [2]. In children, visual axis obstruction during the sensitive period of visual maturation may cause deprivation amblyopia, making timely correction a developmental imperative rather than an elective procedure [3,4].

Ptosis encompasses a broad etiologic spectrum. Acquired disease is most often aponeurotic, but neurogenic, myogenic, mechanical, and traumatic forms are well recognized [1]. Congenital ptosis, usually related to levator dysgenesis, presents different challenges because poor levator function, facial asymmetry, and amblyopia risk shape both timing and surgical goals [4]. Ptosis may also occur within multisystem genetic disorders, in which associated ocular and developmental abnormalities may alter surgical priorities and how surgical success is defined [5,6]. Procedure selection is similarly heterogeneous; for example, levator advancement or resection, Müller’s muscle-conjunctival resection (MMCR), frontalis suspension, and frontalis advancement differ in indication, mechanism, tissue plane, and dependence on levator function [2]. Preoperative selection for internal ptosis repair also varies, including differences in phenylephrine concentration and interpretation of the phenylephrine response [7,8].

Despite the frequency of ptosis repair, no shared definition of surgical success exists. Studies vary in the outcomes they measure, the thresholds they apply, and the follow-up intervals they report. The relative weight assigned to visual function, eyelid height, contour, symmetry, patient satisfaction, recurrence, and reoperation also differs widely [9]. This variation limits comparison across studies and makes apparent differences in surgical effectiveness difficult to interpret.

This review examines six domains of success after ptosis surgery: anatomical, functional, aesthetic, patient-reported, safety, and durability outcomes. We identify the principal sources of heterogeneity across outcome domains and outline priorities for future standardization to improve the measurement, comparison, and communication of surgical benefit.

## 2. Literature Search and Selection

Relevant literature was identified through PubMed/MEDLINE searches from database inception through May 2026, using terms related to blepharoptosis, ptosis surgery, levator advancement, levator resection, Müller’s muscle-conjunctival resection, MMCR, frontalis suspension, surgical outcomes, success criteria, recurrence, and patient-reported outcomes. Reference lists of relevant articles were also reviewed. We focused on English-language peer-reviewed articles relevant to outcome assessment after ptosis surgery, particularly those that defined surgical success or described the outcome measures used. Studies were selected to illustrate the range of outcome definitions in the literature. 

## 3. Anatomical Outcome Measures

### 3.1. MRD1 Thresholds and Cross-Study Comparability

Margin reflex distance 1 (MRD1), defined as the distance between the corneal light reflex and the upper eyelid margin in primary gaze, remains the dominant anatomical endpoint in ptosis surgery [9]. Its appeal lies in its simplicity and familiarity, but its use as a marker of surgical success is inconsistent. 

Published series apply different postoperative MRD1 thresholds across ptosis subtype, operative technique, and composite definitions of success. In acquired ptosis, McCulley et al. defined an ideal result as MRD between 2.0 mm and 4.5 mm with no more than 1 mm of intereyelid asymmetry, achieved in 77% of patients [10]. Dan et al. used an MRD1 threshold of 2.5 mm or greater after MMCR and reported success in 65.7% [11]. Other studies have used postoperative ranges of 3 to 5 mm or 4 to 5 mm, often combined with symmetry or contour criteria [12,13]. In congenital ptosis, Ho et al. defined excellent eyelid height as MRD of 3 mm or greater, achieved in 74.6% of eyelids, while the overall surgical success rate was 97.2% [14]. Thus, a postoperative MRD1 of 2.5 mm may be classified as successful in one study, incomplete in another, and unsuccessful in a third, despite identical central eyelid height. Reported success rates may therefore reflect outcome definitions as much as surgical technique, case mix, or surgeon performance [10,11,14].

Measurement technique introduces further variability. MRD1 may be measured clinically, from standardized photographs, or by digital image analysis, each with different sources of error. Photographic measurements are sensitive to acquisition technique, including patient positioning, lighting, exposure, and camera and flash configuration. Frontalis recruitment can also elevate the upper eyelid and alter measured MRD1, such that studies using the same postoperative threshold may be measuring different physiological states. Standardized photography and automated analysis may improve reproducibility [15,16].

MRD1 also has an inherent construct limitation. It measures central eyelid height but does not capture the broader anatomical result. Eyelid height, contour, and intereyelid symmetry may improve together (Figure 1), but these outcomes do not always align. A patient may meet a prespecified MRD1 threshold while still having clinically relevant abnormalities in eyelid shape, periocular symmetry, dynamic function, or ocular surface exposure. Upper eyelid surgery may also alter lower eyelid position; MRD2 increased by 0.51 mm after MMCR, whereas no significant change occurred after blepharoplasty [17].

### 3.2. Eyelid Contour, Crease Height, and Peak Position

Contour criteria alter reported success because they capture aspects of eyelid position that MRD1 alone cannot measure, including notching, peaking, flaring, and overall contour symmetry (Figure 2) [18]. Matsuda et al. showed this empirically, with success falling from 70.7% using the functional success criteria to 62.2% when contour symmetry, pretarsal show laterality, and eyelid height laterality were required together [19]. Other studies used composite anatomical definitions that incorporated satisfactory contour, interlid crease height, interlid MRD difference, and interlid show difference [12,13]. These differences in endpoint construction limit direct comparison between studies using height-only criteria and those requiring broader anatomical symmetry. Satisfactory eyelid elevation after frontalis suspension may also coexist with residual contour asymmetry (Figure 3).

Crease height and peak position add further variation because they influence eyelid definition, symmetry, and patient-perceived appearance but are not captured by central eyelid height alone. Postoperative descent of the eyelid crease may therefore produce residual asymmetry despite satisfactory MRD1 and eyelid contour (Figure 4). This is especially relevant in East Asian patients, for whom supratarsal crease formation and double-fold morphology are important aesthetic endpoints [20]. Yet these features are rarely measured with standardized, ptosis-specific methods. Contour irregularity, crease asymmetry, and peak malposition may therefore be included in some composite definitions, omitted in others, or recorded as subjective complications, leaving reported success partly dependent on which anatomical details each study chooses to count. Anatomical outcome reporting should therefore specify the MRD1 criterion used and whether contour, symmetry, and crease position were assessed separately or as part of a composite definition.

## 4. Dynamic Function, Frontalis Compensation, and Ocular Surface Safety

Ptosis repair changes brow position, eyelid movement, and ocular surface exposure, yet most outcome studies emphasize static eyelid height in primary gaze. Brow elevation is a common compensatory response to ptosis and often decreases after successful repair; however, postoperative brow position is rarely measured. Brow descent may reduce the perceived gain in eyelid elevation, while persistent frontalis recruitment may leave forehead strain and visual effort unresolved despite an acceptable MRD [21,22]. Wu-Fienberg et al. found that all 28 patients with unilateral horizontal forehead lines had ipsilateral ptosis [23], and Ben Simon et al. described persistent paradoxical frontalis use after anatomically successful repair, with some patients requiring botulinum toxin to suppress the habituated motor response [24]. These findings support frontalis compensation as a patient-relevant outcome, not merely a preoperative sign.

Eyelid function is dynamic, but most studies assess it in primary gaze. Downgaze, blinking, and voluntary closure are important aspects of dynamic eyelid function and ocular surface protection, particularly after frontalis suspension, where eyelid movement depends heavily on brow recruitment rather than physiological levator excursion (Figure 5) [25,26,27]. Conversely, continued habitual frontalis recruitment may produce apparent postoperative overcorrection, which can resolve spontaneously as the patient adapts after surgery (Figure 6). Histologic evidence of proprioceptive sensory fibers in Müller’s muscle further suggests that eyelid position may depend partly on neurosensory feedback rather than mechanical forces alone [28].

Video-based kinematic analysis and automated tracking can quantify blink velocity, palpebral aperture change, and incomplete closure, but these measures remain largely absent from routine outcome reporting [29,30]. Lagophthalmos and exposure keratopathy are important safety outcomes after frontalis suspension; van Sorge et al. reported exposure keratopathy in 26% of eyes after silicone frontalis suspension, although major corneal complications were uncommon [31,32]. Yoon et al. showed that Bell’s phenomenon protects the ocular surface after frontalis suspension, while lagophthalmos greater than 5 mm may increase exposure risk in patients with poor Bell’s phenomenon [33].

Dry eye morbidity should be reported as a procedure-specific safety outcome rather than treated as a general effect of ptosis surgery. For example, Zloto et al. found increased OSDI scores and corneal staining after MMCR at short-term follow-up, with objective dry-eye signs persisting at long-term follow-up, but no comparable change after upper blepharoplasty alone [34,35]. By contrast, levator advancement does not appear to worsen objective tear parameters at short follow-up [36], and transient dry eye worsening typically resolves to near baseline by six months [37]. These findings support ocular surface assessment as a distinct outcome domain, with clear definitions, timing, thresholds for clinical significance, and reporting stratified by procedure type. 

## 5. Symmetry and Aesthetic Outcomes

### 5.1. Inter-Eyelid Symmetry

Symmetry is central to perceived outcome, but most studies define it narrowly as intereyelid MRD1 difference, commonly using thresholds of 1 mm or less [10,11,38,39]. This cutoff has not been validated against patient perception in ptosis populations. A given height difference may be acceptable when contour, brow position, and crease morphology are harmonious (Figure 7), but conspicuous when lid peak position or contour is distorted. 

Height and symmetry should be reported as related but distinct outcomes. Dan et al. reported MRD1 success in 65.7% of operated eyelids and symmetry success in 82.9% of the cohort [11]. Leung et al., in a prospective multicenter MMCR study, similarly separated MRD1 success from symmetry success, defining these as postoperative MRD1 of 2.5 mm or greater and intereyelid MRD1 difference of 1 mm or less, respectively; they reported MRD1 success in 72.1% of operated eyelids and symmetry success in 75.7% of patients [38].

Contralateral ptosis unmasking adds further ambiguity. Under Hering’s law of equal innervation, unilateral ptosis repair may reduce compensatory drive to the fellow levator and reveal subclinical ptosis [40,41]. A technically successful repair may therefore produce new postoperative asymmetry. Studies should specify whether this finding is treated as surgical failure, complication, or expected physiological response, because the distinction affects both outcome reporting and counseling [42].

### 5.2. Aesthetic Assessment Methods

Aesthetic outcome cannot be reduced to intereyelid height difference, yet methods for assessing appearance remain poorly standardized. Aesthetic findings may also vary with assessment timing; transient postinflammatory hyperpigmentation after eyelid surgery appeared within 1–4 weeks and resolved or nearly resolved within 3–5 months in a small case series [43]. In a systematic review of upper blepharoplasty, Hollander et al. found that studies used patient-reported outcomes, scar assessment, eyebrow height, tarsal platform show, and panel or expert evaluation, with sufficient heterogeneity to preclude meta-analysis [44]. Standardized frontal photographs can document eyelid height, contour, crease position, and symmetry, but they provide only a partial representation of periocular appearance [45]. Blinded aesthetic review is also difficult to compare across studies because image acquisition, observer composition, observer training, rating scales, and success thresholds are rarely uniform. Some studies further embed aesthetic criteria within composite success definitions without specifying how those criteria were judged [46]. Patient-reported appearance should not be inferred from observer-rated aesthetic success and is addressed separately below.

Automated landmark detection and image analysis may improve consistency by quantifying eyelid position, contour, and periocular symmetry, and deep-learning models may eventually support comparison with normative datasets [15,18,47]. However, these tools quantify anatomical or observer-defined aesthetic outcomes and should not be interpreted as measures of patient-perceived benefit. These tools remain limited by heterogeneous methods, unvalidated clinical thresholds, and the absence of ptosis-specific outcome platforms. Accordingly, aesthetic findings should be reported as a distinct outcome domain rather than imposed as a universal criterion for surgical success, particularly because their relevance varies by procedure and surgical intent.

## 6. Functional Outcomes Beyond the Superior Visual Field

### 6.1. Visual Field Assessment in Adults

Superior visual field testing is commonly used in adult ptosis surgery to quantify functional impairment and support clinical or administrative decision-making [48]. However, no consensus exists on the optimal perimetric method for measuring ptosis-related impairment [49]. Tangent screen, Goldmann, and Humphrey protocols are used inconsistently across centers, and testing may be shaped as much by reimbursement requirements as by patient-centered functional assessment [49,50].

Measured field loss is also sensitive to eyelid position during testing. Variation in whether, when, and how the eyelid is elevated can change the apparent degree of impairment, even when underlying anatomy is similar [51].

Perimetry captures superior field obstruction under standardized test conditions, but it does not fully represent daily function, including reading in downgaze, driving, mobility, or awareness of the overhead environment. Federici et al. found that superior field and eyelid position predicted postoperative improvement, but preoperative self-reported functional status was the strongest predictor of benefit [52]. Perimetry remains important for documenting functional impairment and may be required for insurance coverage. Patient-reported function provides complementary information when assessing surgical benefit.

### 6.2. Visual Development and Amblyopia in Pediatric Ptosis

In congenital ptosis, functional success is defined by visual development as much as eyelid elevation. After silicone sling suspension, MRD1 improvement and reoperation rates were similar in simple and complex pediatric ptosis, although deliberate undercorrection may be appropriate when reduced Bell’s phenomenon increases exposure risk [53]. The immediate priority is visual axis clearance, particularly in severe unilateral ptosis during early childhood, but long-term success also depends on whether surgery prevents amblyopia without introducing recurrent ptosis or exposure morbidity.

In a population-based cohort study spanning 46 years, 14.9% of children had amblyopia and 40.4% had postoperative lagophthalmos after ptosis repair [54]. Among 84 children undergoing levator resection for unilateral congenital blepharoptosis, reported surgical success was 84.5%, but amblyogenic ametropia was the leading cause of amblyopia, and age at surgery was significantly associated with visual outcome [55]. Anatomical correction alone therefore does not define pediatric success.

Pediatric outcome reporting should therefore be development-specific rather than a direct extension of adult anatomical frameworks. Studies should report visual axis clearance, visual acuity, amblyopia, refractive status, strabismus, recurrence, lagophthalmos, and follow-up through visual maturation. Eyelid-height thresholds alone may overstate success when the primary indication is preservation of visual development.

## 7. Patient-Reported Outcomes: The Missing Center of the Evidence Base

### 7.1. The Discordance Between Anatomical Correction and Patient-Perceived Benefit

Anatomical correction does not reliably predict patient-perceived benefit. In 46 patients undergoing blepharoptosis repair or upper eyelid blepharoplasty, improvement in vision-related quality of life did not correlate with change in upper eyelid MRD [56]. David et al. found high postoperative Glasgow Benefit Inventory scores after MMCR, but preoperative MRD1 was not significantly correlated with postoperative patient-reported benefit [57].

This discordance is clinically plausible. Ptosis affects superior visual field and reading comfort, but also facial expression, social confidence, perceived tiredness, and self-image. In qualitative work, Richards et al. found that patients described appearance-related anxiety, social avoidance, and reluctance to seek treatment because concern about appearance could be perceived as vanity [58]. After surgery, patients described gains in vision, appearance, and psychosocial wellbeing. In subsequent prospective work, successful ptosis surgery reduced appearance-related social distress, anxiety, and fear of negative evaluation, with 85% of patients reporting improved wellbeing [59]. Clinician-defined anatomical success and patient-perceived benefit are therefore related but not equivalent.

### 7.2. Available Instruments and Their Limitations

Existing instruments capture only part of the ptosis experience. The Glasgow Benefit Inventory has been used to measure patient-reported benefit after ptosis surgery, but it was developed for otolaryngology populations and does not assess ptosis-specific appearance, brow strain, contour, or ocular surface symptoms [57,60]. The FACE-Q Eye Module has stronger psychometric grounding and greater relevance to periocular appearance, but it was developed in cosmetic rather than ptosis-specific populations and may show ceiling effects [61,62].

This limitation is clinically important. Although derived from a blepharoplasty population rather than a ptosis population, postoperative patient-reported outcomes explained 47% of the variance in treatment satisfaction, compared with 8% explained by postoperative clinician-rated outcomes [63]. Vision-related quality-of-life measures document functional gains after surgery but are not tailored to ptosis-specific outcomes [64]. Custom questionnaires identify clinically relevant symptoms, but lack the validation and comparability needed for use across studies [65].

### 7.3. Pediatric and Proxy-Reported Outcomes

Patient-reported assessment remains limited in pediatric ptosis. Existing studies have relied on retrospective clinical records or generic validated child- and parent-reported instruments rather than ptosis-specific pediatric measures [66,67]. Pediatric surgery is justified not only by eyelid height or amblyopia risk, but also by psychosocial wellbeing and health-related quality of life [67]. Until age-appropriate and proxy-reported measures are developed, pediatric outcome assessment will remain disproportionately dependent on clinician-reported endpoints. 

### 7.4. The Unmet Need for a Ptosis-Specific Instrument

No validated instrument currently addresses the functional, aesthetic, psychosocial, brow-related, and ocular surface burden of ptosis [56,57,58,60,61,62,63,64,65,68,69,70]. A ptosis-specific instrument should be developed from patient-derived domains, including visual function in daily activities, appearance-related self-perception, psychosocial distress, brow and forehead burden, and ocular surface symptoms. Patient involvement is essential because objective clinical outcomes may not fully capture patient-perceived benefit [59]. Patient-reported outcomes should complement, rather than replace, objective anatomical measures. Patient dissatisfaction with an appearance-related feature does not by itself indicate technical failure of ptosis repair, but it may identify an outcome not captured by eyelid height alone. Without such a measure, assessment of patient benefit will continue to depend largely on anatomical variables that capture only part of the postoperative experience.

## 8. Durability, Recurrence, and Reoperation

### Assessing Whether Surgical Success Persists

Early postoperative success does not establish durability. Reported follow-up varies widely, from a few weeks to as long as 10 years, though most studies assess outcomes within the first 6 months [14,36,71]. Eyelid height, contour, ocular surface status, and patient satisfaction may all change after this period.

Durability is better assessed with time-to-event methods than with binary success at a single postoperative visit. In 319 patients with congenital ptosis, Kaplan–Meier analysis showed recurrence-free rates of 97.3%, 80.5%, and 76.7% at 1, 5, and 10 years after levator resection, compared with 90.9%, 42.9%, and 20.8% after frontalis suspension [14]. In adults, Cohen et al. reported recurrence in 23% of eyes over a median follow-up of 165 weeks and identified non-aponeurotic ptosis as the only independent predictor [71].

Recurrence is inconsistently defined. Cohen et al. defined recurrence as a decrease of at least 1 mm in MRD after initially successful repair, whereas Ke et al. defined recurrence as postoperative MRD1 below 1 mm [71,72]. These definitions capture different events and could produce different recurrence rates in the same population. Early residual ptosis should be distinguished from later recurrence after an initially successful repair; the postoperative timepoint separating these outcomes is not standardized across studies. Late eyelid descent may also reflect progressive tissue or age-related change rather than failure of the original repair.

Reoperation is an imperfect surrogate for failure. Revision may reflect undercorrection, recurrent ptosis, overcorrection, contour abnormality, lagophthalmos, asymmetry, or patient dissatisfaction. An excellent eyelid position at the conclusion of levator resection in a child may still be followed by overcorrection on the first postoperative day requiring revision surgery (Figure 8). Chou et al. reported an overall reoperation rate of 8.7% among 1519 patients, with undercorrection the leading indication [73]. Conversely, absence of reoperation may reflect patient preference, surgeon threshold, access to care, or reluctance to undergo further surgery rather than durable success. Technique and implant material also influence durability; recurrence after silicone rod suspension has been associated with rod migration, reduced tensile strength, and micromorphological degradation [74], while banked irradiated [75] and lyophilized fascia lata [76] suspensions have shown late recurrence during extended follow-up. Long-term studies should report follow-up duration, recurrence definitions, reoperation indications, material-related complications, and time-to-event analyses when follow-up intervals vary.

## 9. Conclusions

Reported success rates in ptosis surgery reflect how success is defined as much as what surgery achieves. Variation in outcome measures, thresholds, and follow-up limits comparison across studies, techniques and patient populations. MRD1, eyelid contour, and symmetry with the fellow eye remain essential, but no single measure captures the full range of surgical outcomes. More consistent reporting is needed to distinguish differences in surgical effectiveness from differences in measurement.

A core outcome set could provide a framework for improving comparability while allowing clinical interpretation to reflect the indication for surgery and the intended treatment goal. Recent surgeon consensus work has identified important variables for ptosis evaluation and provides a foundation for this effort [9]. The goal is not a single universal definition of success, but consistent reporting that preserves clinical context. Table 1 identifies priorities for further standardization toward that aim.

## Figures and Tables

**Figure 1:**
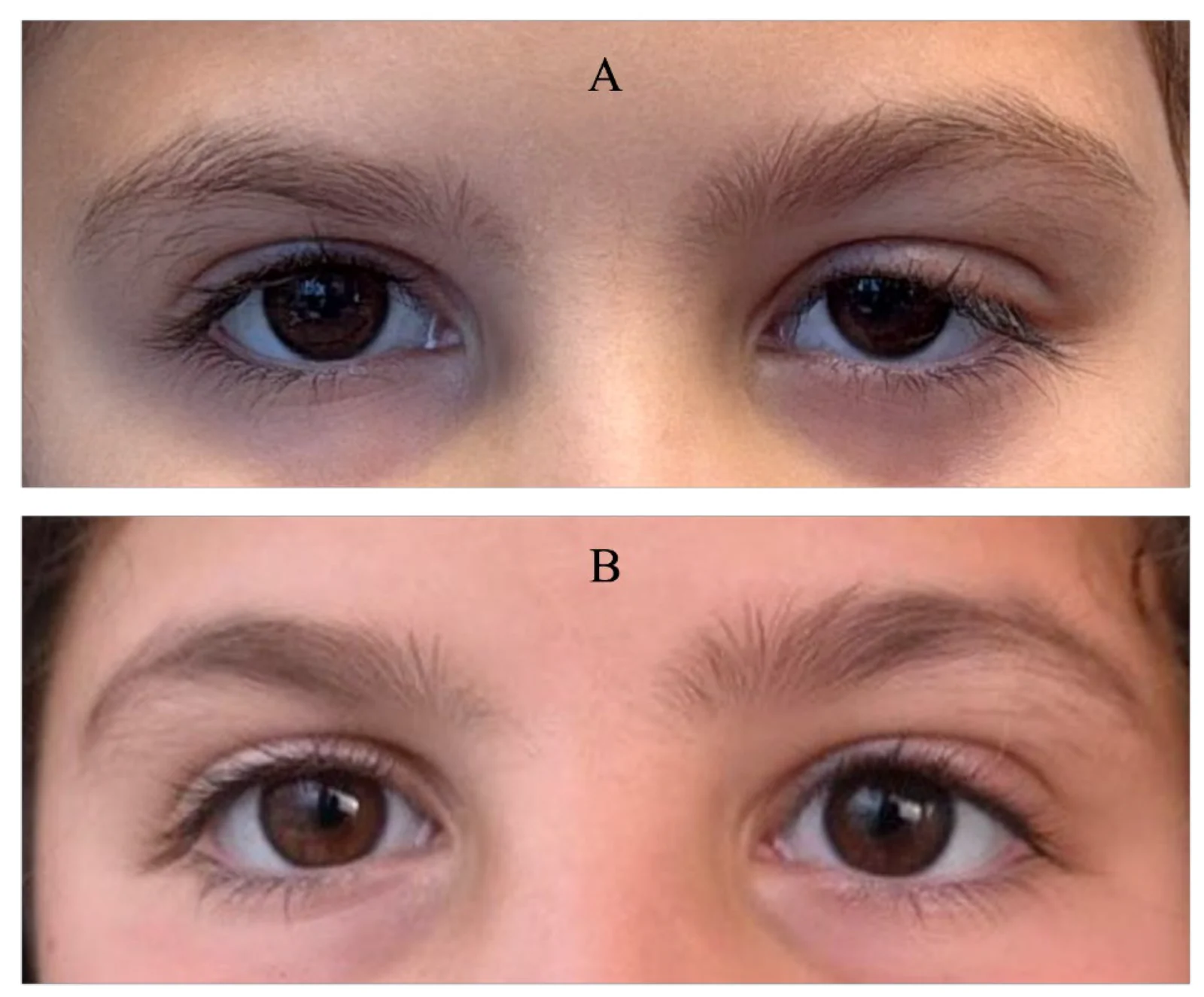
Satisfactory anatomical outcome after levator resection. Preoperative ((**A**), upper) and postoperative ((**B**), lower) photographs following left levator resection, demonstrating improvement in upper-eyelid height, contour, and intereyelid symmetry. MRD1 of the left eye improved from 2 mm preoperatively to 4 mm postoperatively.

**Figure 2:**
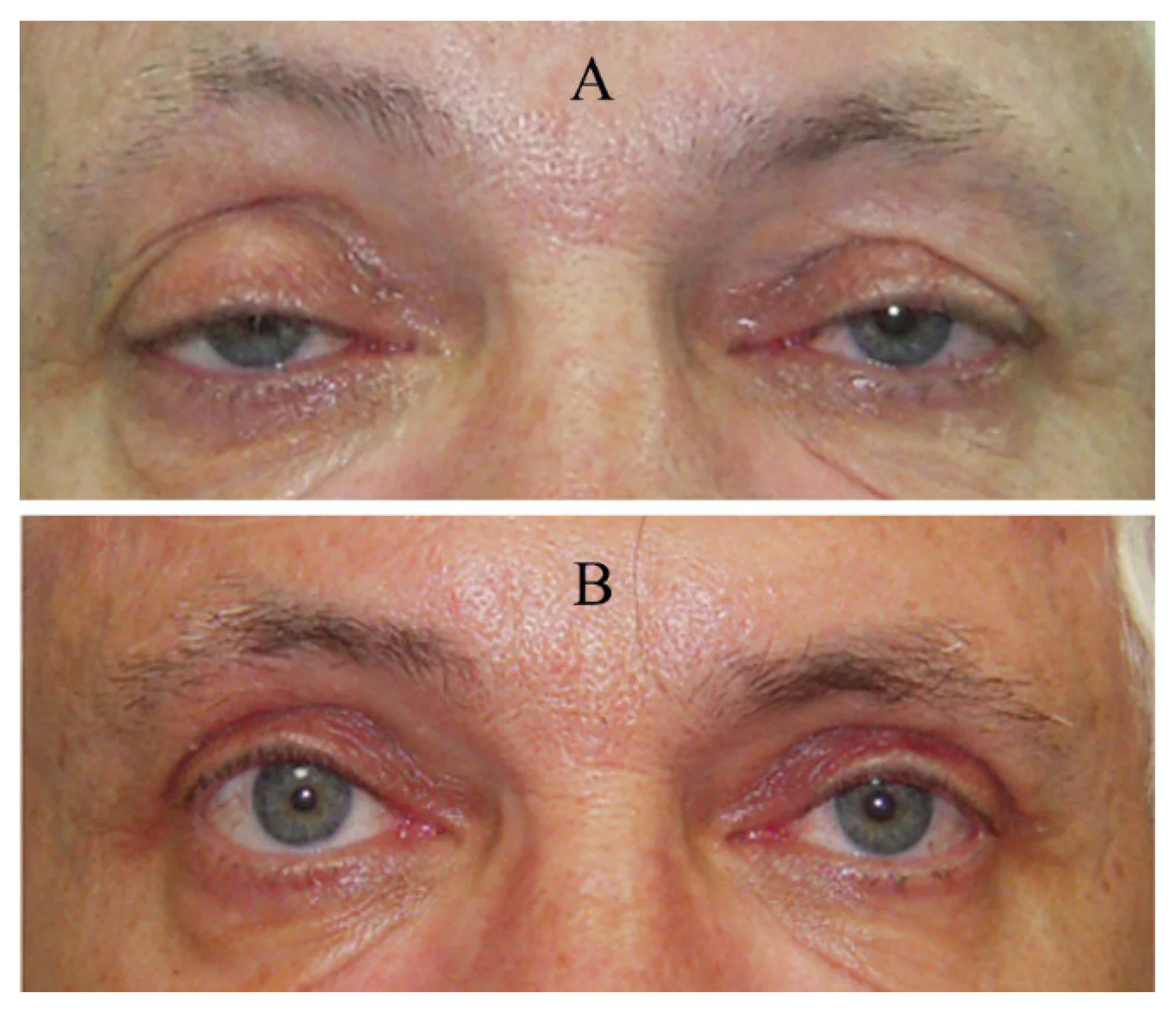
Residual medial ptosis following levator advancement. Preoperative (**A**) and postoperative (**B**) photographs demonstrating satisfactory improvement in eyelid height but mild residual medial ptosis, resulting in an imperfect eyelid contour.

**Figure 3:**
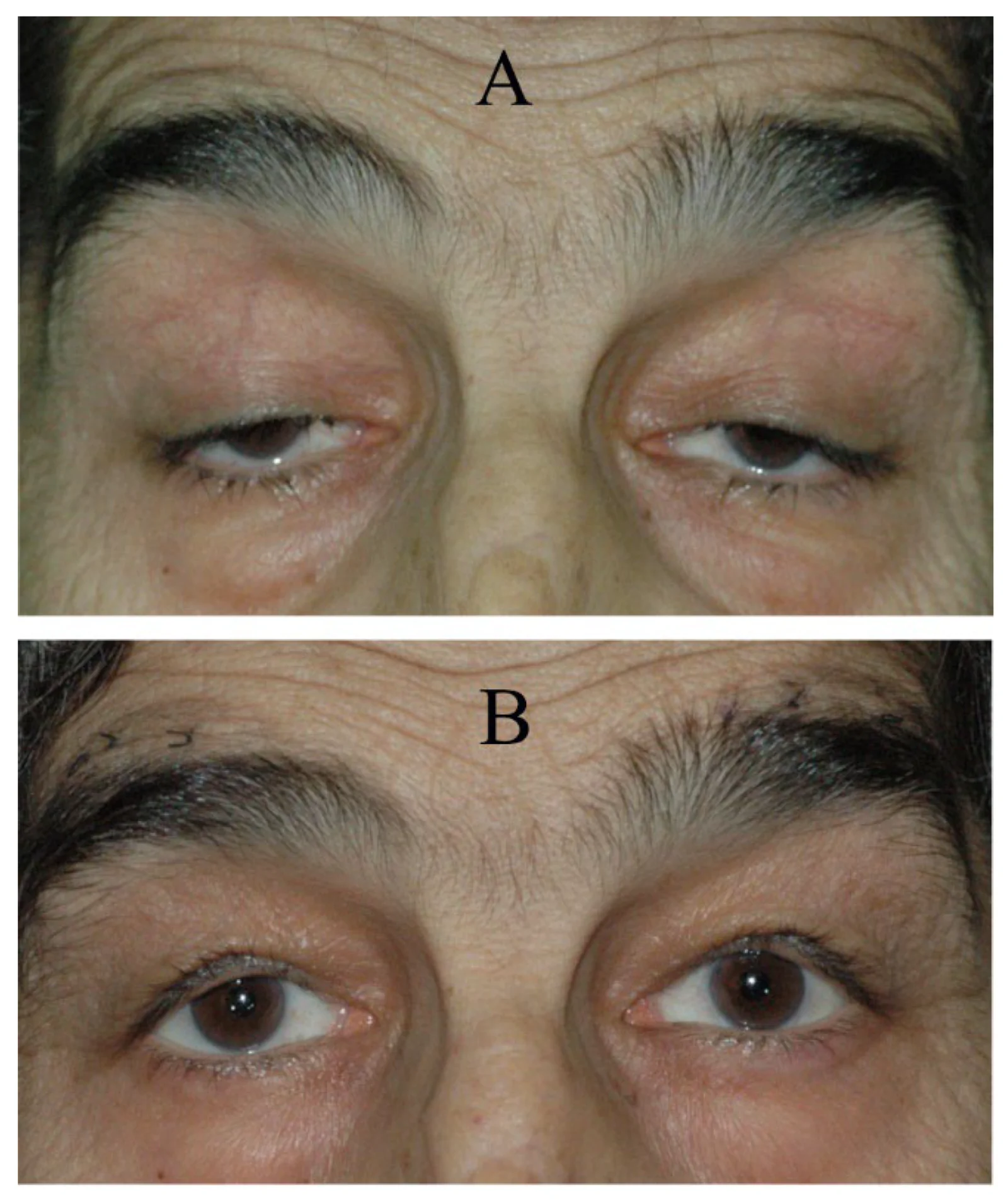
Residual contour asymmetry after frontalis suspension. Preoperative (**A**) and postoperative (**B**) photographs demonstrating satisfactory postoperative eyelid elevation but persistent asymmetry of the eyelid contour.

**Figure 4:**
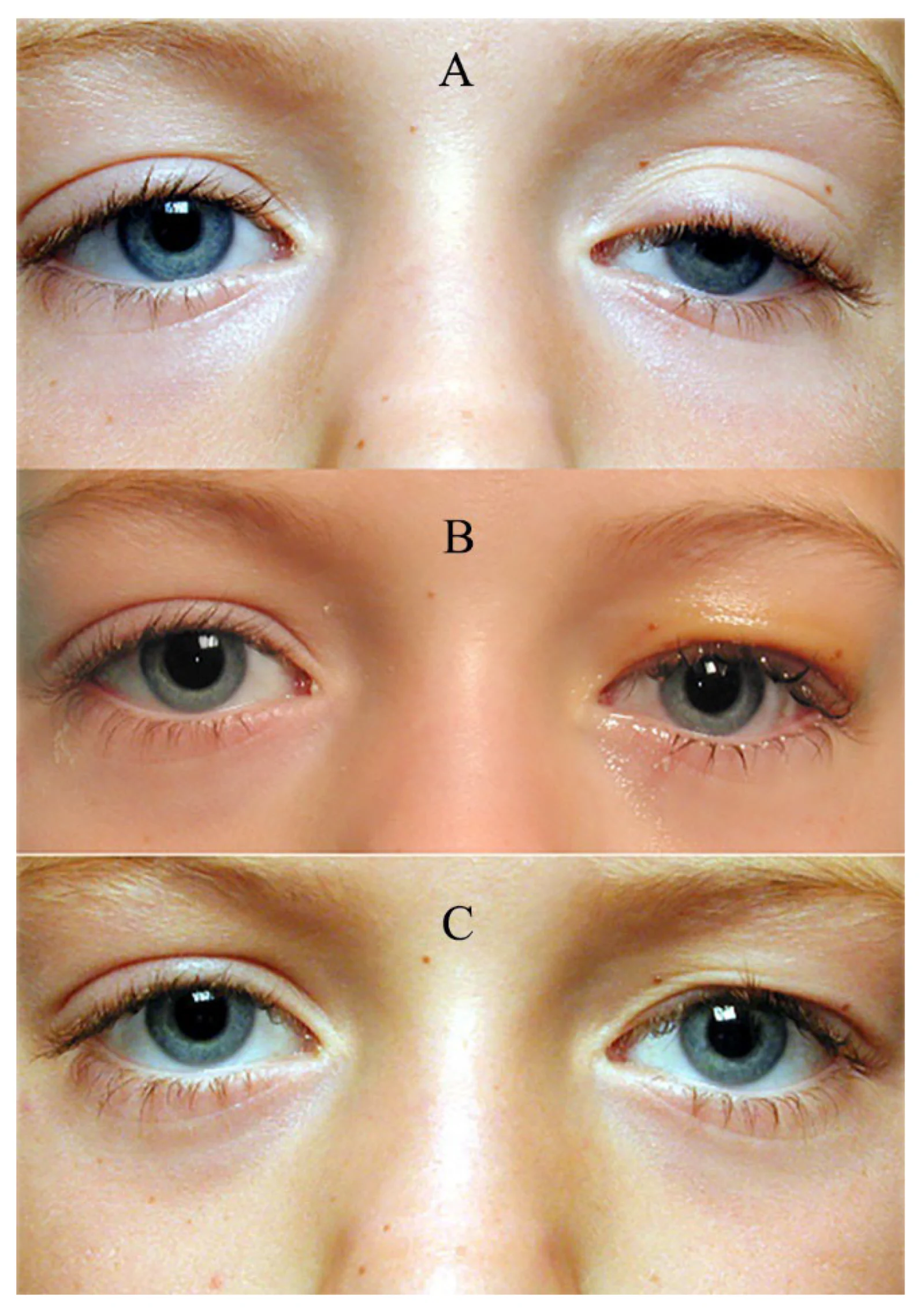
Residual eyelid-crease asymmetry after levator resection. Preoperative (**A**) and postoperative (**B**,**C**) photographs demonstrating satisfactory MRD1 and eyelid contour after levator resection, with residual asymmetry caused by inferior displacement of the eyelid crease.

**Figure 5:**
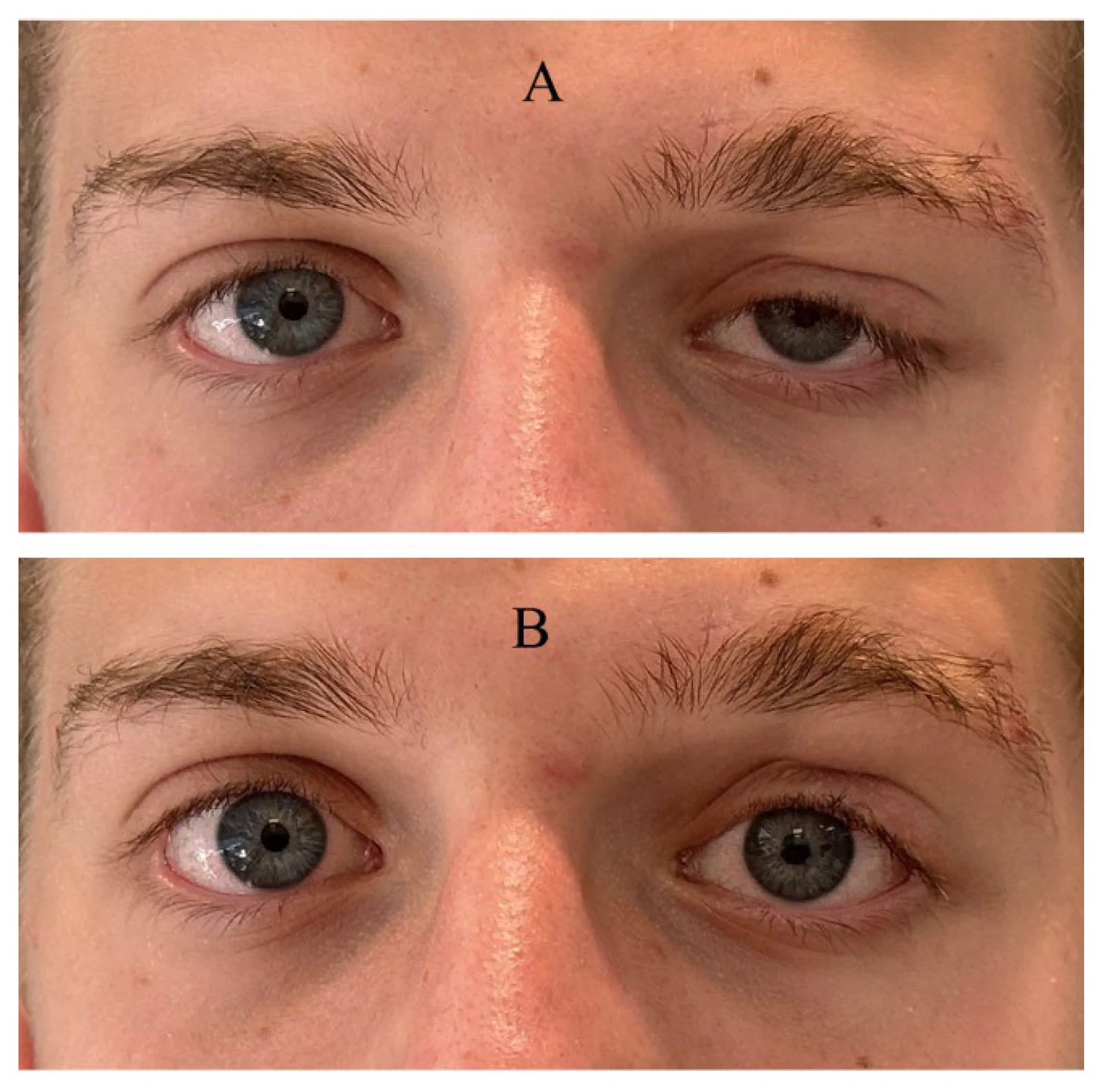
Effect of brow recruitment on eyelid height after frontalis suspension. Without active brow elevation (**A**), postoperative MRD1 appears low and could be interpreted as undercorrection. With active brow elevation (**B**), MRD1 becomes symmetric, demonstrating that the functional result after frontalis suspension depends partly on postoperative brow recruitment.

**Figure 6:**
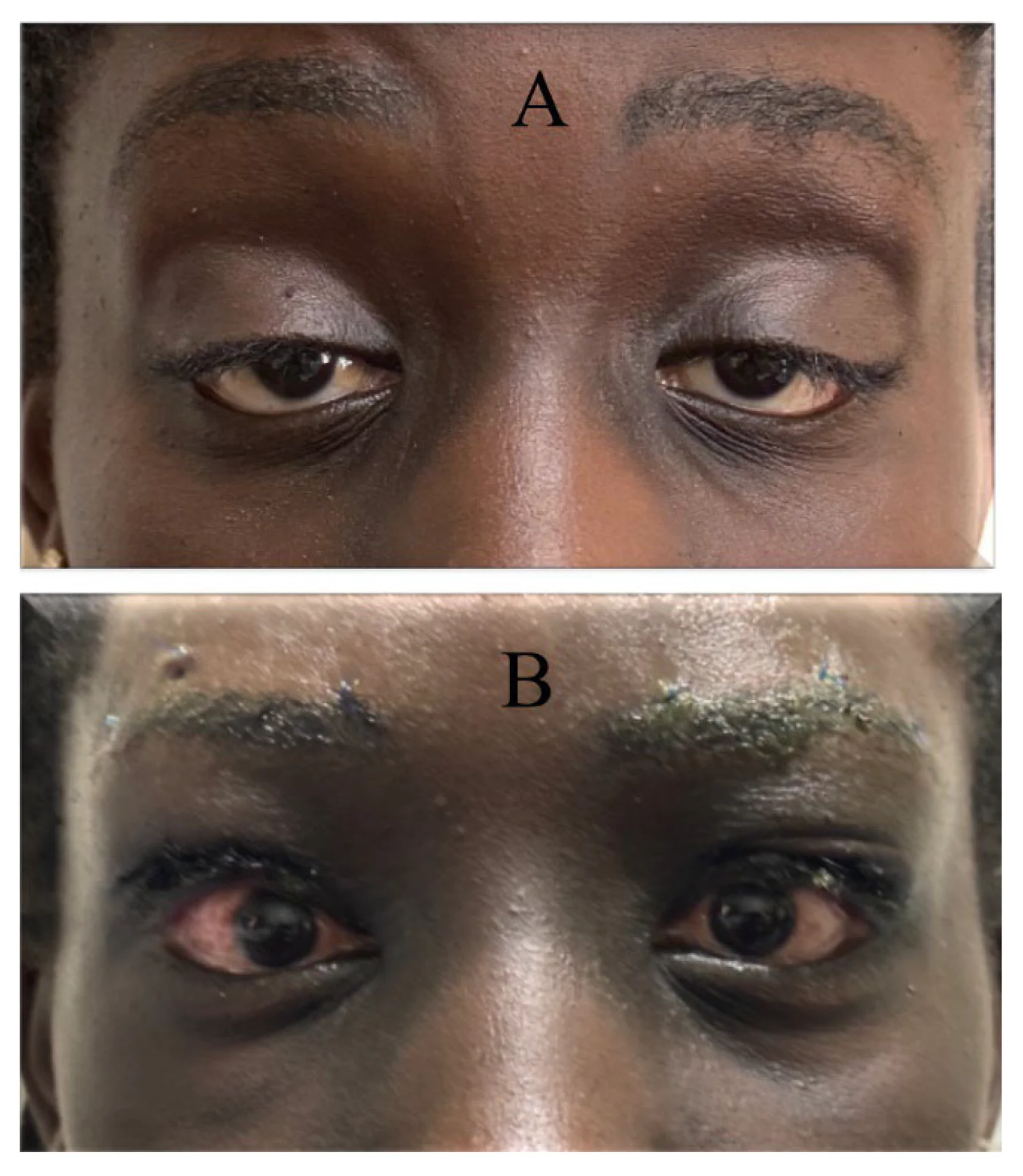
Apparent overcorrection caused by paradoxical frontalis use after frontalis suspension. Preoperative (**A**) and postoperative (**B**) photographs demonstrating postoperative overcorrection associated with continued habitual recruitment of the frontalis muscle. The overcorrection improved spontaneously over time as frontalis recruitment subsided, without the need for botulinum toxin treatment.

**Figure 7:**
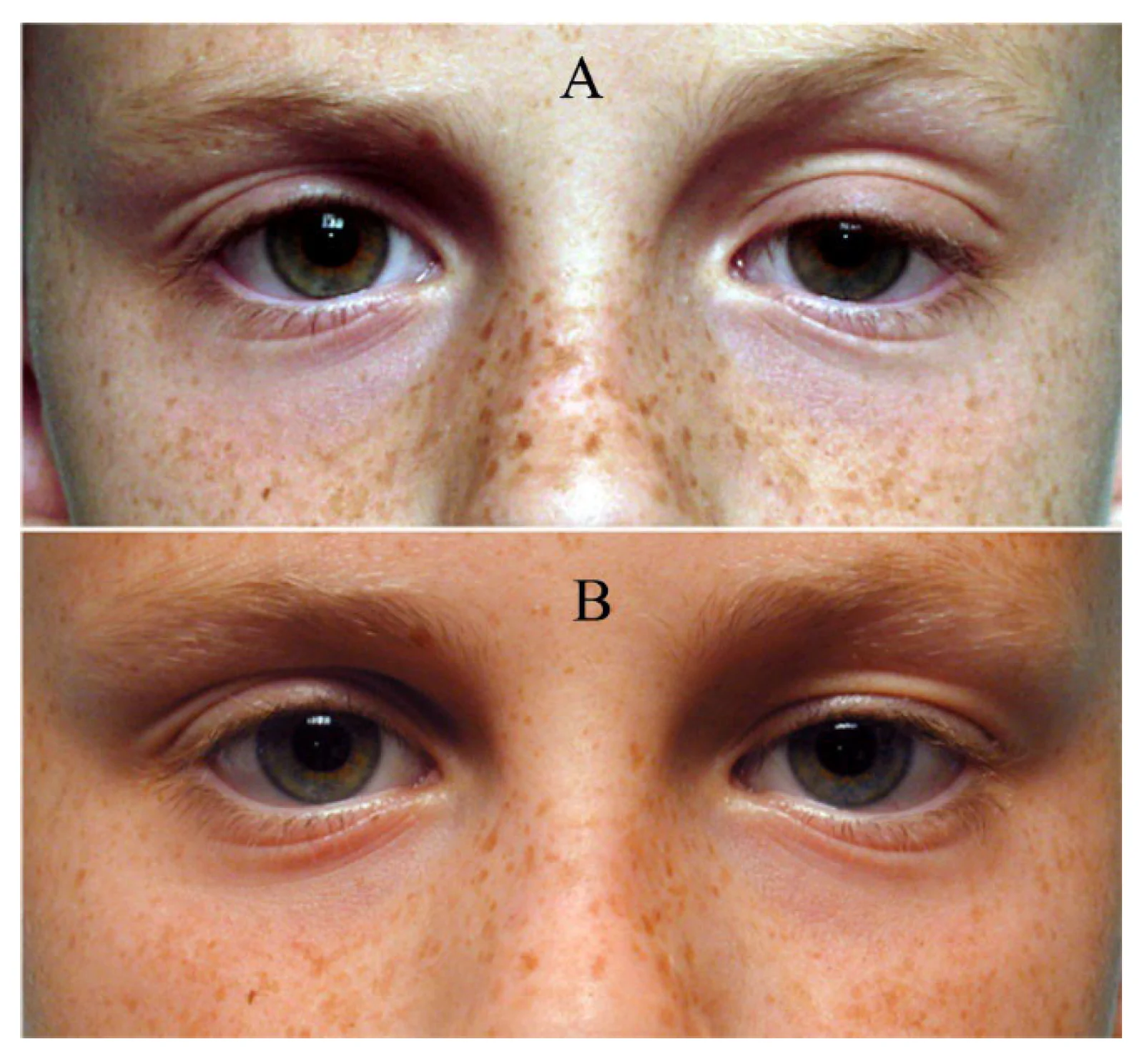
Residual MRD1 asymmetry despite a satisfactory postoperative appearance. Preoperative (**A**) and postoperative (**B**) photographs following left levator resection, demonstrating an attractive eyelid contour and satisfactory overall result despite mild residual intereyelid asymmetry. Postoperatively, MRD1 was 4 mm in the right eye and 3.5 mm in the left eye, corresponding to an intereyelid difference of 0.5 mm.

**Figure 8:**
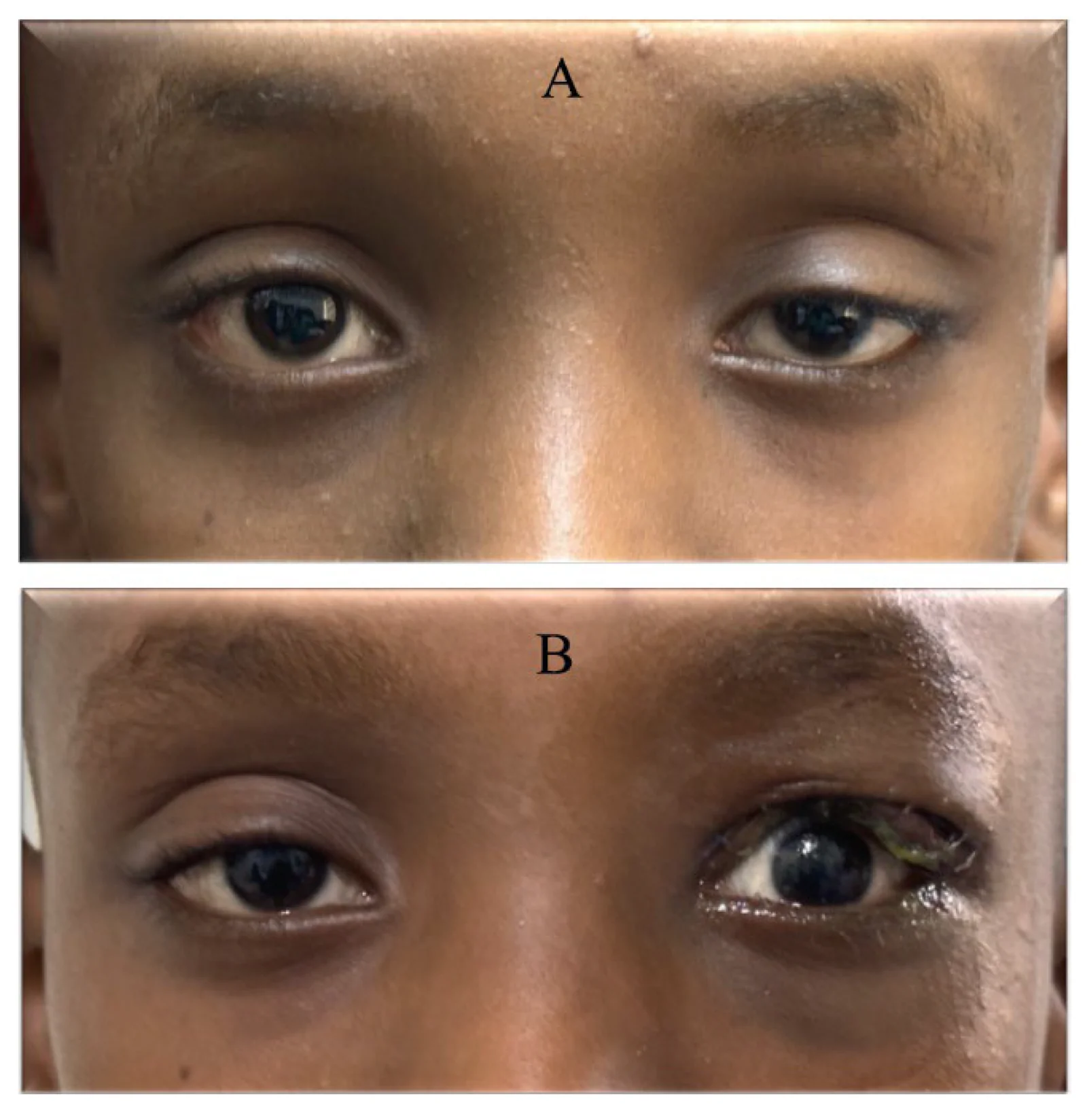
Early overcorrection after levator resection. Preoperative photograph of a child with unilateral ptosis (**A**). Despite excellent eyelid position at the conclusion of levator resection, marked overcorrection was present on the first postoperative day (**B**) and subsequently required surgical revision.

**Table 1:** Priority Outcome Domains for Standardized Reporting in Ptosis Surgery. The relevance of individual domains may vary by indication, procedure, and patient population. The priorities below are intended as a clinically informed agenda for standardization rather than a validated classification or consensus recommendation.

Priority	Unresolved Question
1. Anatomical definitions	Which MRD1 thresholds and additional anatomical criteria, including contour, symmetry, crease height, and peak position, should be reported separately or included in composite definitions? Should these criteria differ by ptosis subtype, surgical technique, or patient age?
2. Functional outcomes	Which measures of visual and dynamic eyelid function should be included, and how should they be standardized across patient populations and procedures?
3. Patient-reported outcomes	Which patient-reported domains are essential to ptosis outcome assessment, and what instrument development is needed to measure them validly across surgical and demographic groups?
4. Ocular surface safety	Which ocular surface parameters should be reported as procedure-specific safety outcomes, at what timepoints, and using what thresholds for clinical significance?
5. Recurrence and durability	How should recurrence and reoperation be defined, and what follow-up duration and analytical methods are needed to assess durability?
6. Pediatric outcomes	What outcome framework is appropriate for pediatric ptosis, where visual development, anatomical correction, and ocular surface safety must be considered together?

## Data Availability

No new data were created or analyzed in this study. Data sharing is not applicable to this article.
